# Deciphering the Role of a SLOG Superfamily Protein YpsA in Gram-Positive Bacteria

**DOI:** 10.3389/fmicb.2019.00623

**Published:** 2019-04-05

**Authors:** Robert S. Brzozowski, Mirella Huber, A. Maxwell Burroughs, Gianni Graham, Merryck Walker, Sameeksha S. Alva, L. Aravind, Prahathees J. Eswara

**Affiliations:** ^1^Department of Cell Biology, Microbiology and Molecular Biology, University of South Florida, Tampa, FL, United States; ^2^National Center for Biotechnology Information, National Library of Medicine, National Institutes of Health, Bethesda, MD, United States

**Keywords:** FtsZ, GpsB, *Bacillus subtilis*, oxidative stress, cell division, NAD, SLOG

## Abstract

Bacteria adapt to different environments by regulating cell division and several conditions that modulate cell division have been documented. Understanding how bacteria transduce environmental signals to control cell division is critical in understanding the global network of cell division regulation. In this article we describe a role for *Bacillus subtilis* YpsA, an uncharacterized protein of the SLOG superfamily of nucleotide and ligand-binding proteins, in cell division. We observed that YpsA provides protection against oxidative stress as cells lacking *ypsA* show increased susceptibility to hydrogen peroxide treatment. We found that the increased expression of *ypsA* leads to filamentation and disruption of the assembly of FtsZ, the tubulin-like essential protein that marks the sites of cell division in *B. subtilis*. We also showed that YpsA-mediated filamentation is linked to the growth rate. Using site-directed mutagenesis, we targeted several conserved residues and generated YpsA variants that are no longer able to inhibit cell division. Finally, we show that the role of YpsA is possibly conserved in Firmicutes, as overproduction of YpsA in *Staphylococcus aureus* also impairs cell division.

## Importance

Although key players of cell division in bacteria have been largely characterized, the factors that regulate these division proteins are still being discovered and evidence for the presence of yet-to-be discovered factors has been accumulating. How bacteria sense the availability of nutrients and how that information is used to regulate cell division, positively or negatively, is less well-understood even though some examples exist in the literature. We discovered that a protein of hitherto unknown function belonging to the SLOG superfamily of nucleotide/ligand-binding proteins, YpsA, influences cell division in *Bacillus subtilis*, directly or indirectly, by integrating nutrient availability and growth rate. We also showed that YpsA is important for the oxidative stress response in *B. subtilis*. Furthermore, we provide evidence that the possible cell division inhibition function of YpsA is also conserved in another Firmicute, *Staphylococcus aureus*. This first report on the role of YpsA brings us a step closer in understanding the complete tool set that bacteria have at their disposal to precisely regulate cell division to adapt to varying environmental conditions.

## Introduction

Cell division in bacteria is a well-orchestrated event that is achieved by the concerted action of approximately a dozen different key division proteins (Haeusser and Margolin, 2016). Amongst them is a protein central to cell division, a tubulin-like protein FtsZ, which marks the site of cytokinesis in most bacteria (Busiek and Margolin, 2015; Du and Lutkenhaus, 2017). In addition to standard spatial regulators of septum positioning (Eswara and Ramamurthi, 2017), factors that sense nutrient availability (Wang and Levin, 2009; Monahan et al., 2014a), DNA damage (Dajkovic et al., 2008; Mo and Burkholder, 2010; Modell et al., 2014), and alternate external environments (Justice et al., 2008; Khandige et al., 2016), have been shown to influence cell division. The observation that cell division in model organisms *Escherichia coli* and *B. subtilis* that lack well-studied Min and nucleoid occlusion regulatory systems undergo cell division largely unperturbed (Monahan et al., 2014b), and other reports that suggested Min system does not play a role in division site selection in *B. subtilis* (Gregory et al., 2008; Eswaramoorthy et al., 2011); prompted us to investigate the presence of other factors involved in cell division regulation. Here we describe the possible role of YpsA, a protein conserved in several members of the Firmicutes phylum, in cell division.

**Figure 1:**
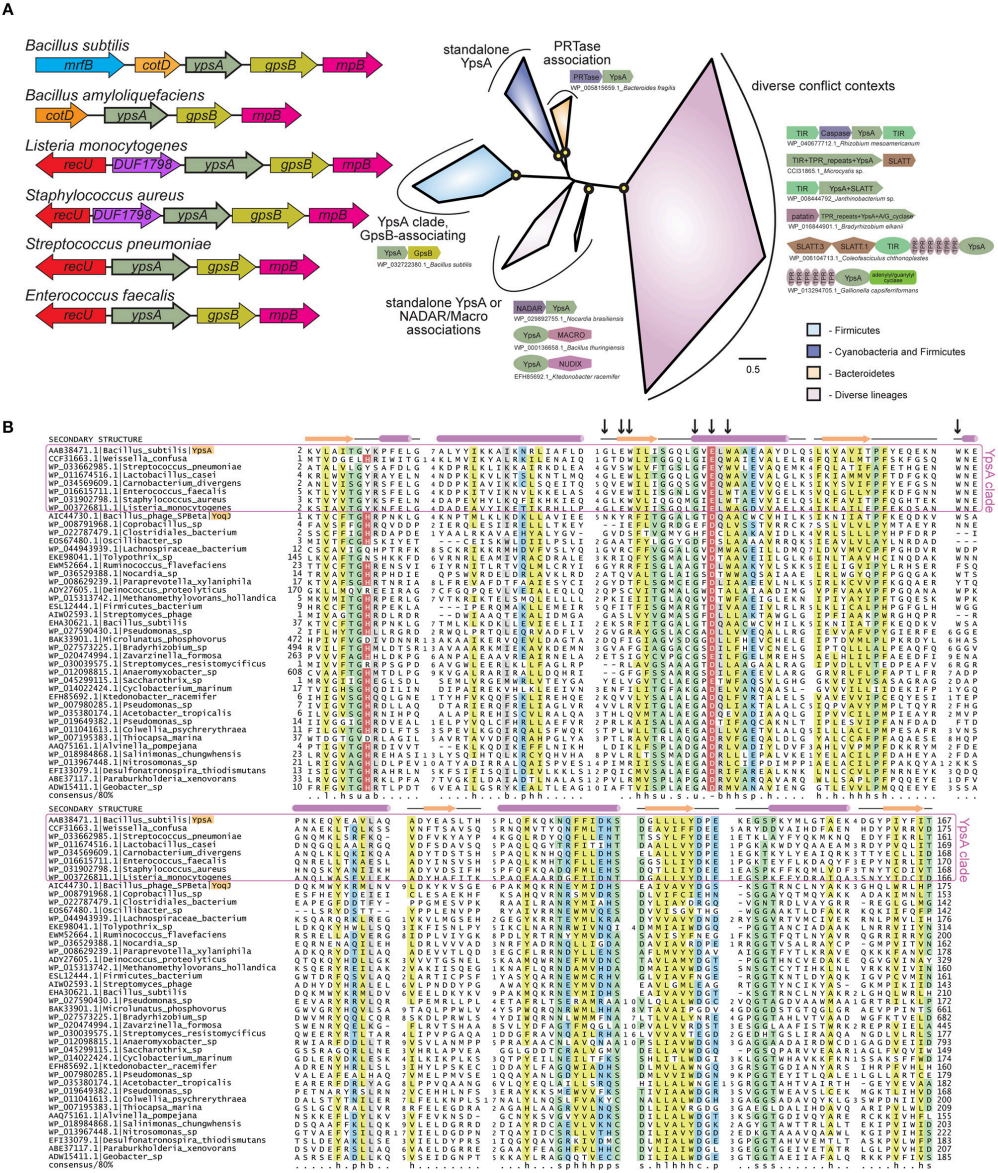
**(A)** Left: Cartoon representation of *ypsA* gene neighborhood in Firmicutes, not to scale. The genes that encode protein products containing a domain of unknown function DUF1798 are named as such. Right: Phylogenetic tree of the YpsA family, key branches with >70% bootstrap support are denoted with yellow circles. Reproducible clades within the family are color-coded according to their phyletic distribution and labeled with names and representative conserved domain architectures and gene neighborhoods. For these genome context depictions, colored polygons represent discrete protein domains within a protein, while boxed arrows represent individual genes within a neighborhood. Each context is labeled with NCBI accession and organism name, separated by an underscore. For gene neighborhoods, the labeled gene contains the YpsA domain. Abbreviations: A/G_cyclase, adenylyl/guanylyl cyclase. **(B)** Multiple sequence alignment of the YpsA family of proteins. Secondary structure and amino acid biochemical property consensus are provided on the top and bottom lines, respectively. Black arrows at top of alignment denote positions subject to site-directed mutagenesis. Sequences are labeled to left with NCBI accession and organism name separated by vertical bars. Gene names from the text are provided after organism name. Selected members of the YpsA clade, which associate with GpsB, are enclosed in a purple box. YpsA and YpsA-like YoqJ are highlighted in orange. Alignment coloring and consensus abbreviations as follows: b, big and gray; c, charged and blue; h, hydrophobic and yellow; l, aliphatic and yellow; p, polar and blue; s, small and green; u, tiny and green. The conserved aromatic position in the first loop, abbreviated ‘a', and the conserved negatively-charged position in the second helix, abbreviated ‘-', are both colored in red with white lettering to distinguish predicted, conserved positions located within the active site pocket.

The genes *ypsA* and *gpsB* (formerly *ypsB*) are in a syntenous relationship in many Firmicute genomes (Figure 1A). GpsB is a cell division protein that regulates peptidoglycan synthesis in *B. subtilis* (Claessen et al., 2008; Tavares et al., 2008), *Streptococcus pneumoniae* (Fleurie et al., 2014; Rued et al., 2017), and *Listeria monocytogenes* (Rismondo et al., 2016). More recently, our group showed that *Staphylococcus aureus* GpsB affects the polymerization kinetics of FtsZ directly (Eswara et al., 2018). As genes in a syntenous arrangement across multiple genomes, often referred to as conserved gene neighborhoods, are commonly indicative of functional relationships (Aravind, 2000; Huynen et al., 2000), we were curious to study the function of YpsA in *B. subtilis*. Prior to our investigation, the crystal structure of *B. subtilis* YpsA was solved by a structural genomics group (PDB ID: 2NX2). Based on the unique structure and sequence features (Figure 1B), YpsA was classified as the founding member of the “YpsA proper” clade in the *S*MF/DprA/*LOG* (SLOG) protein superfamily (Burroughs et al., 2015). The SLOG superfamily contains a specific form of the Rossmannoid fold, and is involved in a range of nucleotide-related functions. These include the binding of low-molecule weight biomolecules, nucleic acids, free nucleotides, and the catalyzing of nucleotide-processing reactions (Fischer et al., 2006; Mortier-Barrière et al., 2007; Samanovic et al., 2015). Recently, several members of the SLOG superfamily were further identified as key components in a newly-defined class of biological conflict systems centered on the production of nucleotide signals. In these systems, SLOG proteins are predicted to function either as sensors binding nucleotide signals or as nucleotide-processing enzymes generating nucleotide derivatives which function as signals (Burroughs et al., 2015). Despite these new reports, the precise function of YpsA and its namesake family have yet to be experimentally investigated.

Here we report that (i) YpsA provides protection against oxidative stress; (ii) overexpression of *ypsA* causes mislocalization of FtsZ-GFP that results in cell filamentation, which is dependent on the growth rate; (iii) YpsA-GFP forms dynamic foci that are possibly mediated by nucleotide binding; and finally (iv) overexpression of *ypsA* in *S. aureus* results in cell enlargement, typical of cell division inhibition in cocci (Pinho and Errington, 2003)—suggesting a conserved function for YpsA across Firmicutes with very different cell-morphologies. In sum, these results constitute the first report on YpsA and its role in oxidative stress response and possibly in cell division regulation.

## Results

### YpsA Provides Oxidative Stress Protection

**Figure 2:**
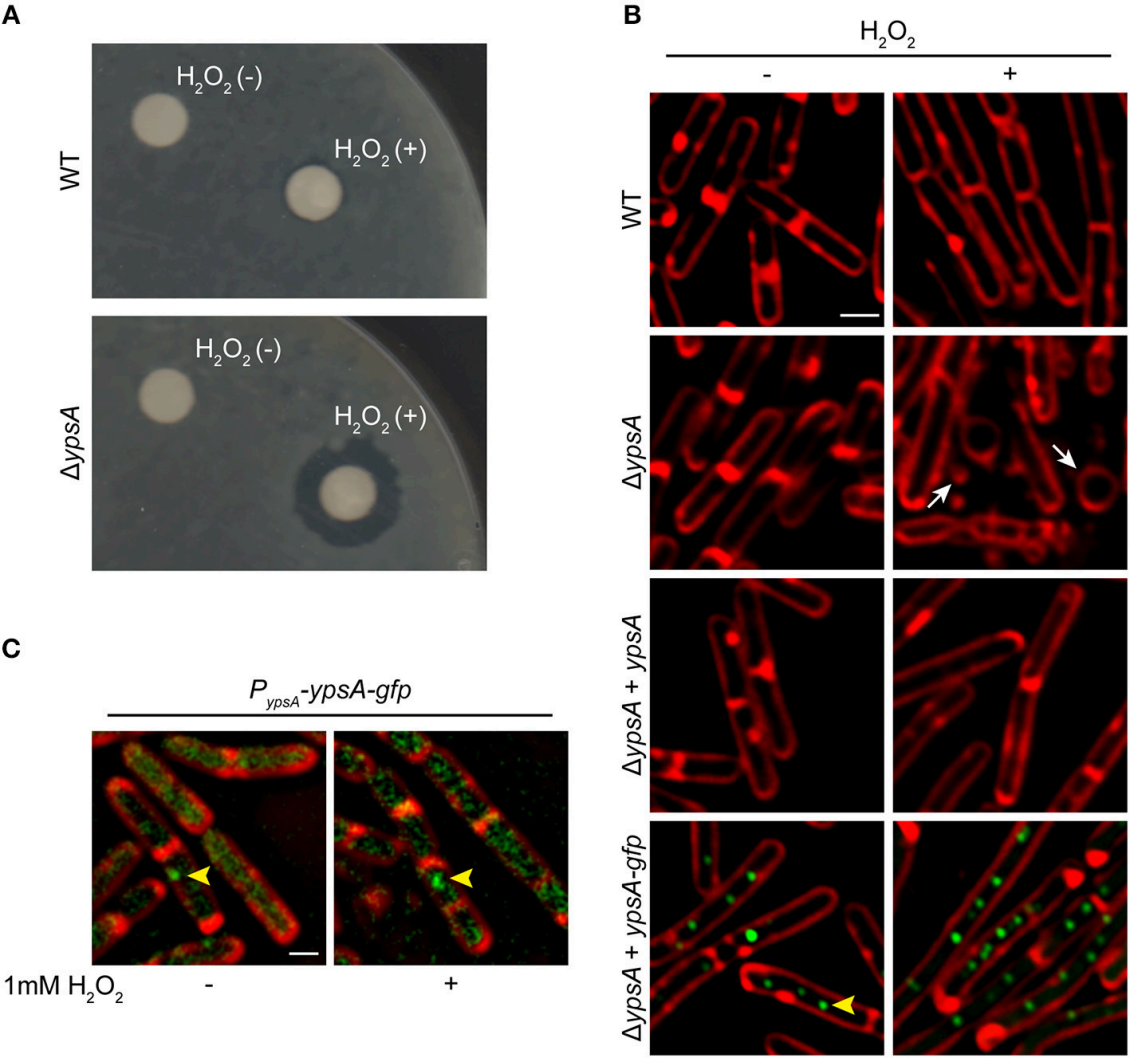
YpsA plays a role in oxidative stress response. **(A)** Disc diffusion assay with lawns made of WT (PY79) or a strain lacking *ypsA* (RB42) treated with blank disc and 1 M H_2_O_2_ are shown. **(B)** Fluorescence micrographs showing cells of WT (PY79), Δ*ypsA* (RB42), and Δ*ypsA* complemented with a copy of inducible *ypsA* or *ypsA-gfp* at an ectopic locus (RB160 or RB161) grown for 1 h with or without 1 mM H_2_O_2_, 250 uM IPTG, and stained with FM4-64 (membrane, red). White arrows indicate aberrantly shaped cells and vesicle-like structures. **(C)** Fluorescence micrographs of RB221 strain that produces YpsA-GFP under the control of its native promoter imaged at mid-log (OD600 = 0.5) phase with or without H2O2 treatment. Yellow arrowheads indicate YpsA-GFP foci. Scale bar: 1 μm.

As a first step to study the significance of YpsA, we studied the phenotype of a *ypsA* null strain in several stress-inducing conditions through a standard disc-diffusion assay with compounds that induce: DNA damage (0.1 mM mitomycin C-treatment), membrane stress (1% SDS or 5 mg/ml daptomycin), general stress (a range of 10–50% ethanol), cell wall stress (commercially available discs with Penicillin 10 units and vancomycin 30 μg), oxidative stress (0.1 M, 0.5 M, or 1 M H_2_O_2_); as well as heat stress (growth of cells at 48°C). We noticed that the *ypsA* null strain exhibited a larger zone of inhibition in comparison to the wild type (WT) control, reproducibly, only when incubated with discs containing 0.5 M or 1 M H_2_O_2_ (WT: 1.8 ± 0.45 mm; Δ*ypsA*: 7.6 ± 0.54 mm; Figure 2A), suggesting that YpsA provides protection against oxidative stress. It is noteworthy that *ypsA* transcript level is elevated upon hydrogen peroxide treatment [(Nicolas et al., 2012; Zhu and Stülke, 2018); Figure S1]. To further evaluate this phenotype, we monitored the cells grown in liquid culture in the absence or presence of 1 mM H_2_O_2_ using fluorescence microscopy (Figure 2B). Untreated cells lacking *ypsA* appear morphologically similar to WT (Figure 2B; see WT and Δ*ypsA* left panels) and grew similar to WT in conditions tested (Figure S3A). Although WT cells were tolerant to H_2_O_2_ treatment, Δ*ypsA* cells displayed obvious signs of a sick phenotype with cells that are en route to lysis with membrane abnormalities, cell morphology changes, and vesicle-like structure formations (Figure 2B; compare WT and Δ*ypsA* panels; see arrows). Quantification of H_2_O_2_-treated cells revealed that 27% of WT and 79% of Δ*ypsA* cells were sick (i.e., number of cells in a representative field of view that showed the sick phenotype described in the previous sentence; *n* = 100). To test if this phenotype is specifically due to absence of YpsA, we introduced an inducible copy of *ypsA* at an ectopic locus. In the presence of inducer, H_2_O_2_-treated cells resemble WT (Figure 2B, see Δ*ypsA* + *ypsA* panel; 23% sick cells, n = 100) indicating that YpsA is responsible for providing protection against H_2_O_2_-induced oxidative stress. Similarly, production of YpsA-GFP from an ectopic locus also provided oxidative stress protection in the presence of inducer suggesting that YpsA-GFP is a functional fusion (Figure 2B, see Δ*ypsA* + *ypsA-gfp* panel; 21% sick cells, *n* = 100).

### Increased Production of YpsA Inhibits Cell Division

**Figure 3:**
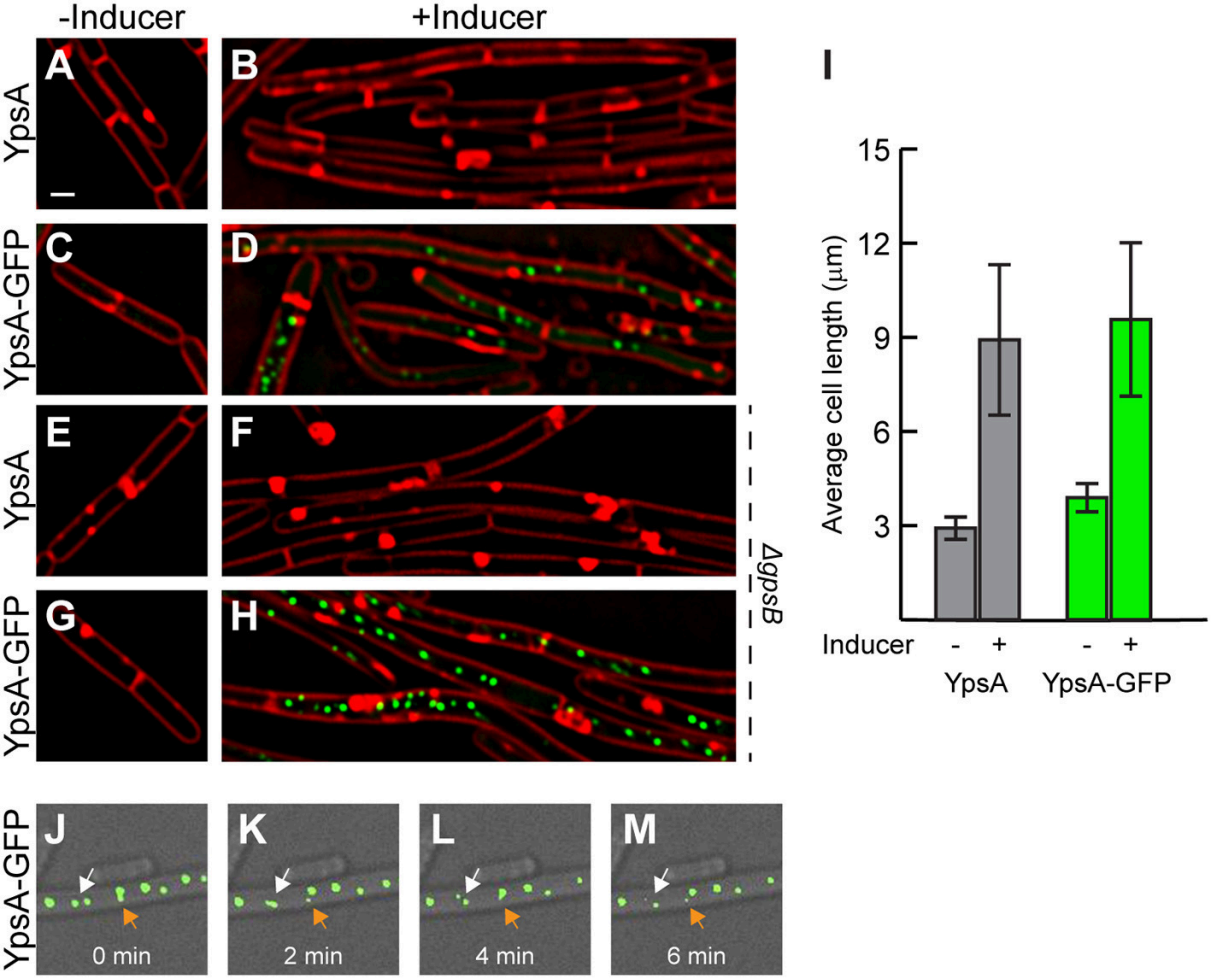
Elevated production of YpsA or YpsA-GFP leads to inhibition of cell division. **(A–D)** Morphology of cells containing inducible *ypsA* (GG82) or *ypsA-gfp* (GG83) grown in the absence of inducer IPTG **(A,C)** or in the presence of inducer for 1 h **(B,D)**. **(E–H)** Cell morphology of strains lacking *gpsB* and containing either inducible *ypsA* (RB43) or *ypsA-gfp* (RB44) grown in the absence **(E,G)** or presence **(F,H)** of inducer. **(I)** Quantification of cell lengths of cells shown in **(A–D)**. Time-lapse micrographs of *ypsA-gfp* expressing cells (GG83) at time intervals indicated at the bottom. White and orange arrows follows different foci that are mobile. DIC (gray) and fluorescence of membrane dye (FM4-64; red), GFP (green) are shown. Scale bar: 1 μm.

Next, we examined the *ypsA* overexpression phenotype. For this purpose, we constructed an otherwise WT-strain to ectopically express either *ypsA* or *ypsA-gfp* upon addition of inducer. Quantification of GFP fluorescence revealed that there was 3-fold overproduction of YpsA-GFP in the presence of inducer (2,415 ± 1,296 arbitrary units; *n* = 50) when compared to YpsA-GFP produced under the control of its native promoter (732 ± 692 arbitrary units; *n* = 50; RB221). We then monitored the cell morphology of cells overproducing YpsA or YpsA-GFP. To our surprise, as shown in Figure 3, when compared to the cell lengths of inducible strains grown in the absence of inducer [YpsA: 2.92 ± 0.81 μm (Figure 3A); YpsA-GFP: 3.89 ± 0.98 μm (Figure 3C); *n* = 100], cells overproducing YpsA or YpsA-GFP appeared elongated [YpsA: 8.92 ± 4.89 μm (Figure 3B); YpsA-GFP: 9.57 ± 4.99 μm (Figure 3D); *n* = 100] implying that cell division is inhibited by YpsA (Figure 3I). This result also indicated that the fluorescent protein tagged fusion of YpsA is functional reaffirming the conclusion derived in the previous section. Tracking fluorescence of YpsA-GFP in our overexpression strain showed that YpsA assembles into discrete foci (Figure 3D). To study whether YpsA is capable of self-interaction, based on the ability to form a focus, we conducted a FLAG tag-based immunoprecipitation assay in a strain producing both YpsA-GFP and YpsA-FLAG. Upon using YpsA-FLAG as a bait we were able to pulldown YpsA-GFP species indicating that YpsA is capable of forming a homocomplex (Figure S2). An unrelated protein, housekeeping sigma factor SigA, served as our negative control which was not present in the eluate fraction. To further analyze the YpsA-GFP foci in our overexpression strain, we conducted time-lapse microscopy at 2 min intervals for 6 min which revealed that YpsA foci are dynamic within a 1 μm range (Figures 3J–M; see arrows; also see the video taken at 1 min intervals in the Supplementary Information). Quantification of the YpsA foci revealed that on average there are 2, 4, or 9 foci per cell in cells that are smaller than 5 μm, between 5 and 10 μm, or larger than 10 μm, respectively (*n* = 100). Also, upon tracking 100 different foci,we observed short-range (<1 μm) mobility in 71% of the cells (*n* = 100). Since YpsA-GFP retains fluorescence as a focus and that the foci are mobile, and focus disruption occurs in some strains that express mutated versions of *ypsA* (Figure 6A), we conclude that the foci are not artifacts of non-functional misfolded aggregates. We also observed that foci formation occurs when YpsA-GFP production is driven from its native promoter and upon H_2_O_2_ treatment in 9% and 12% of the cells, respectively (Figure 2C).

Since genes coding for YpsA and the cell division protein GpsB are in a syntenous relationship, we aimed to test whether the YpsA overproduction-mediated filamentation is dependent on GpsB. As shown in Figures 3E–H, cells lacking *gpsB* also formed filaments upon overexpression of *ypsA* or *ypsA-gfp*, suggesting that YpsA-mediated cell division inhibition is independent of GpsB.

### YpsA Overproduction Disrupts FtsZ Assembly

**Figure 4:**
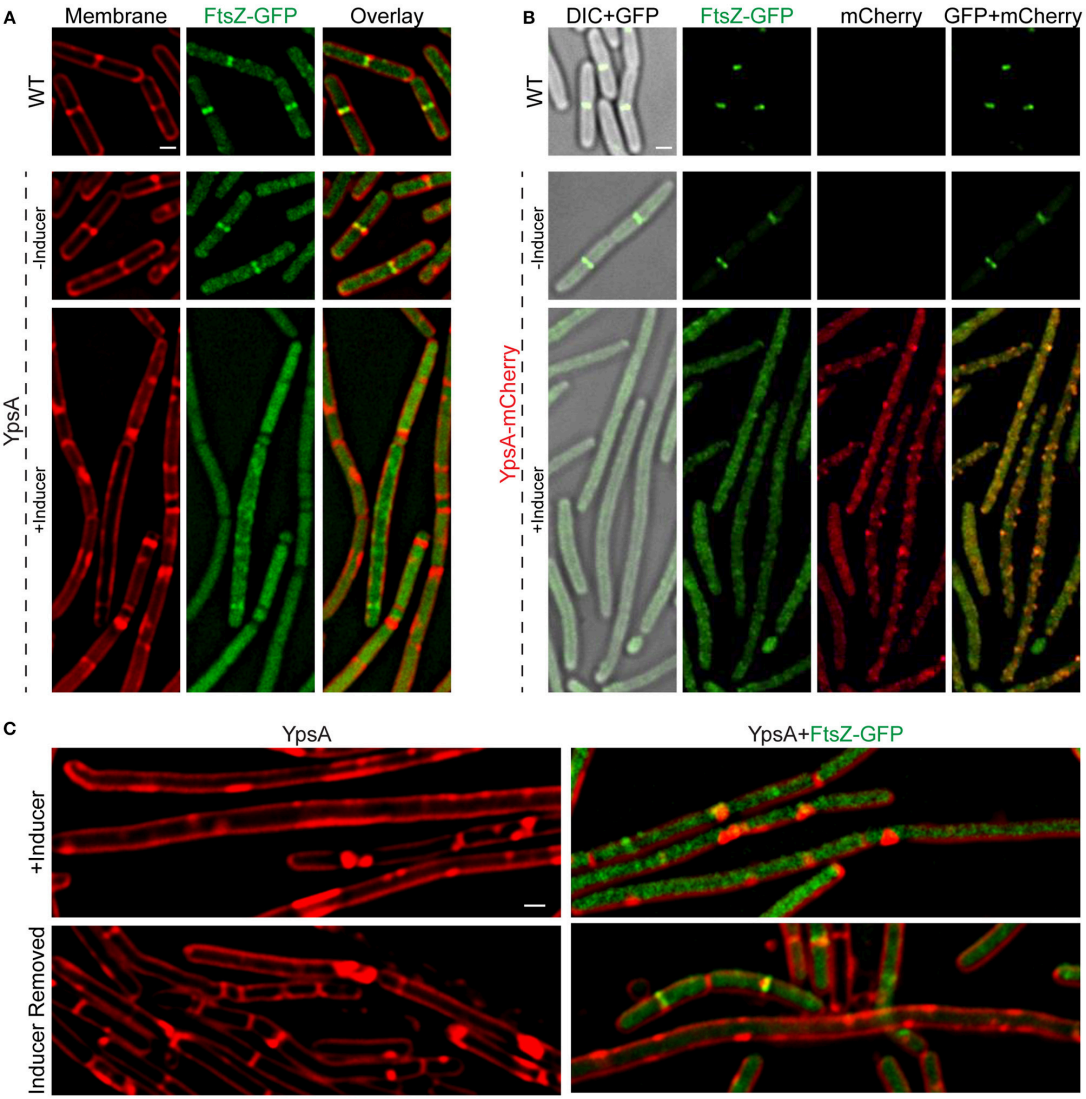
YpsA inhibits FtsZ ring assembly. **(A)** Fluorescence micrographs of cells that either constitutively produce FtsZ-GFP in otherwise wild type strain (PE92; top panel) and cells that constitutively produce FtsZ-GFP and additionally harbor a copy of inducible *ypsA* (RB15) grown in the absence (middle panel) or presence of inducer IPTG are shown. Fluorescence of FM4-64 membrane dye (red) and GFP (green) are shown. **(B)** Cellular morphologies of cells that constitutively produce FtsZ-GFP (PE92) and cells that additionally contain a copy of inducible *ypsA-mCherry* (RB97) grown in the absence (middle panel) or presence of inducer are shown. DIC (gray) and fluorescence of GFP (green) and mCherry (red) are shown. **(C)** Cells of GG82 (inducible *ypsA*) and RB15 (inducible *ypsA* + constitutively expressed *ftsZ-gfp*) were imaged after grown in the presence of inducer for 1 h (top panels) or 3 h after the removal of inducer (bottom panels). Scale bars: 1 μm.

Typically, filamentation is a result of impaired FtsZ ring assembly. To test whether FtsZ ring assembly is affected by YpsA overproduction, we engineered strains that constitutively produces FtsZ-GFP (Gregory et al., 2008; Eswaramoorthy et al., 2011), to also produce either *ypsA* or *ypsA-mCherry* under the control of an inducible promoter. In FtsZ-GFP producing otherwise WT cells, the cell length appeared normal and FtsZ assembled into FtsZ rings at mid-cell in 90% of the cells (Figures 4A,B; top panels; *n* = 100). In the strains capable of producing both FtsZ-GFP and YpsA or YpsA-mCherry, when cells were grown in the absence of inducer, FtsZ-GFP localization appeared similar to the control strain (Figures 4A,B; middle panels). In striking contrast, when cells were grown in the presence of inducer FtsZ-GFP assembled into rings in only 8% or 3% of cells that express *ypsA* or *ypsA-mCherry*, respectively (Figures 4A,B; bottom panels; *n* = 100).

### Resumption of Cell Division After Removal of Inducer

Next, we wished to test whether filamentous cells resume normal division following the removal of inducer. For this purpose, we grew the cells of the inducible *ypsA* strain with or without the FtsZ-GFP reporter until the mid-logarithmic phase (OD_600_ = 0.5). We then added the inducer to induce the production of YpsA. Monitoring the cell morphology of these cells 1 h post addition of the inducer, revealed filamentation in cells expressing *ypsA* (cell length: 9.17 ± 4.76 μm; Figure 4C top left panel; *n* = 100). In cells that also produce the FtsZ-GFP reporter, in addition to filamentation (cell length: 8.20 ± 5.17 μm; Figure 4C top right panel), we also observed disruption of the FtsZ-ring assembly (FtsZ rings: 11% in cells <5 μm and 4% in cells ≥5 μm). We then pelleted the cell culture and washed off the inducer with a fresh medium thrice. Following the growth of the cells in fresh medium, 3 h after the removal of the inducer, we observed that the cell lengths of *ypsA* expressing cells with or without the FtsZ-GFP reporter were 4.30 ± 2.14 μm and 5.66 ± 4.40 μm, respectively (Figure 4C bottom panels), indicating restoration to normal growth. Quantification of FtsZ rings revealed resumption of FtsZ ring assembly subsequent to the removal of inducer (FtsZ rings: 57% in cells <5 μm and 7.5% in cells ≥5 μm; Figure 4C bottom right panel). These observations suggest that normal division resumes in filamentous cells after the removal of the inducer.

### Sporulation Frequency Is Unaffected in *ypsA* Strains

Since *cotD*, which codes for a spore coat protein, is immediately upstream of *ypsA* (Figure 1A), we were curious to see if *ypsA* has any role in sporulation. To address this, we performed a sporulation assay using Casein Hydrolysate (CH)-based growth medium and Sterlini-Mandelstam sporulation medium in triplicates (Eswaramoorthy et al., 2009). The average sporulation frequency of Δ*ypsA* strain was 176% relative to WT (100%), which is a modest 2-fold increase in frequency, suggesting YpsA has no appreciable role in sporulation. To study whether YpsA overproduction-mediated filamentation impairs sporulation, we conducted a similar sporulation assay and found that cells overexpressing *ypsA* (98%) or *ypsA-gfp* (127%) also displayed a sporulation frequency similar to WT.

### YpsA Overproduction-Induced Filamentation Is Dependent on Growth Rate

**Figure 5:**
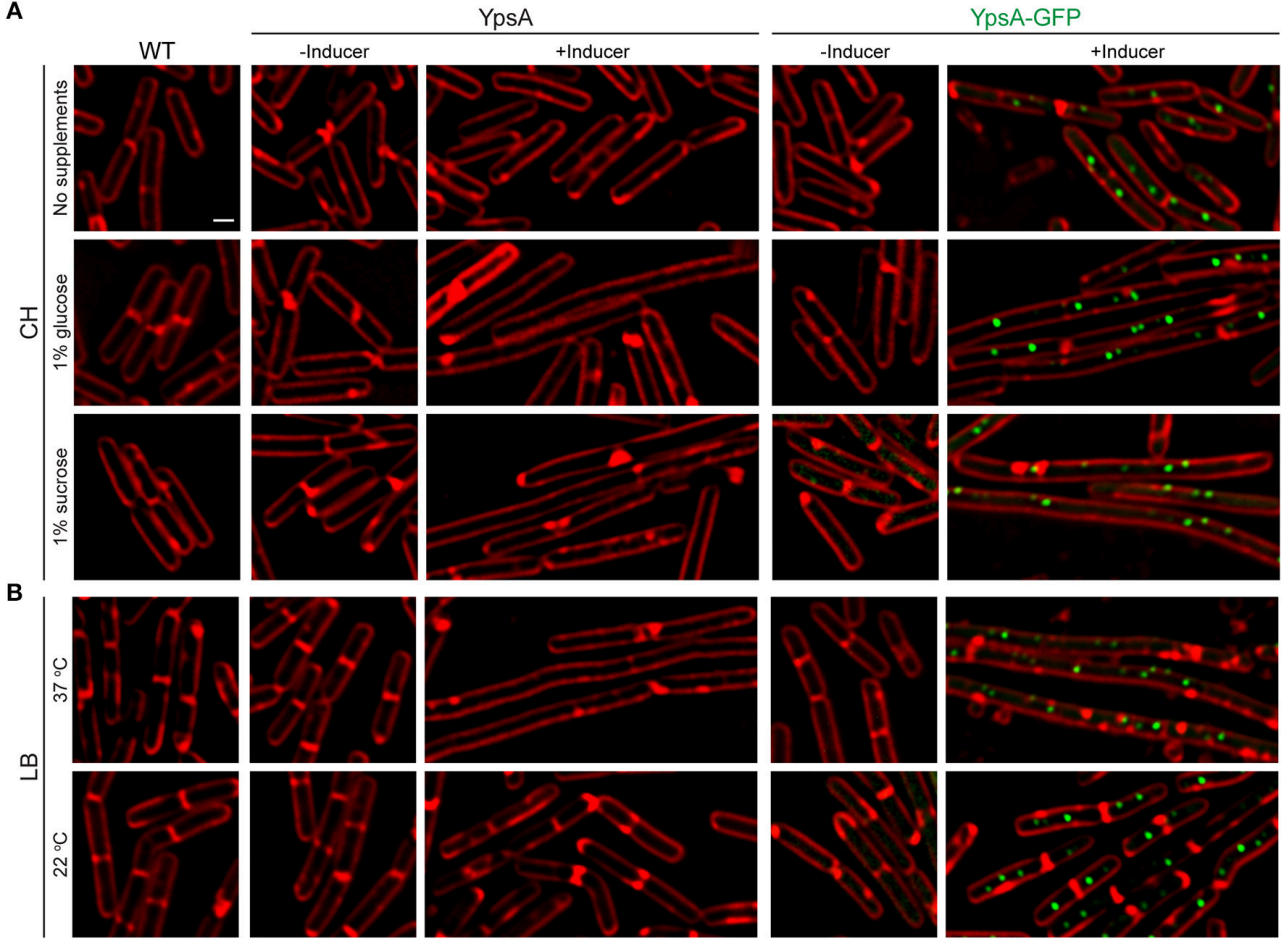
Growth rate-dependent inhibition of cell division. **(A)** Fluorescence micrographs of cells of WT, inducible *ypsA* (GG82) or *ypsA-gfp* (GG83) were grown in CH medium in the absence of any supplements (top panels), with 1% glucose supplementation (middle panels), or with 1% sucrose supplementation (bottom panels). **(B)** WT, GG82, or GG83 strains described above were grown in LB medium at 37°C (top panels) or 22°C (bottom panels). Fluorescence of membrane dye (FM4-64; red), GFP (green) are shown. Scale bar: 1 μm.

To fully comprehend how filamentous cells achieve WT-like sporulation efficiency, we observed the cell morphology of *ypsA* overexpressing cells grown in the presence of inducer in CH medium using fluorescence microscopy. Surprisingly, the cell lengths of *ypsA* or *ypsA-gfp* overexpressing cells appeared similar to WT when grown with or without the inducer (YpsA: 2.73 ± 0.69 μm vs. 3.17 ± 0.80 μm; YpsA-GFP: 3.02 ± 0.68 μm vs. 3.15 ± 0.84 μm; *n* = 100; Figure 5A top panels), unlike what we observed when cells were grown in LB medium (compare Figure 5A top panels with Figure 5B top panels). In the presence of inducer in *ypsA-gfp* expressing cells, although cells were not filamentous, YpsA-GFP foci formation was still observed (Figure 5A top right panel).

We hypothesized that the lack of nutrients in CH compared to LB might be the reason for the lack of filamentation. To test our hypothesis, we externally supplemented either 1% glucose or 1% sucrose to the CH medium. Intriguingly, mid-log phase cells (OD_600_ = 0.5) grown in CH in the presence of glucose and inducer to overproduce YpsA or YpsA-GFP lead to filamentation (YpsA: 3.14 ± 0.65 μm vs. 7.93 ± 2.39 μm; YpsA-GFP: 3.02 ± 0.84 μm vs. 10.31 ± 5.26 μm; *n* = 100; Figure 5A middle panels). Similar results were observed when the CH medium was supplemented with sucrose (YpsA: 2.94 ± 0.64 μm vs. 9.53 ± 2.69 μm; YpsA-GFP: 2.87 ± 0.78 μm vs. 9.71 ± 3.51 μm; *n* = 100; Figure 5A bottom panels), suggesting that filamentation is dependent on nutrient availability.

To evaluate whether the filamentation is linked to nutrient availability or perhaps the growth rate, we monitored the growth rate by measuring the absorbance at 600 nm (OD_600_) over the course of 5 h. We observed that supplementation of either glucose or sucrose increased the growth rate when compared to strains grown in CH medium without any supplementation (Figure S3B). As an independent analysis to study this phenomenon of growth rate-dependent cell division inhibition, we grew cells in LB medium at either 22 or 37°C. As expected, cultures grown at 22°C displayed a slower growth rate compared to cultures grown at 37°C (Figure S3C). Next, we imaged the mid-log phase (OD_600_ = 0.5) cells of cultures grown at 22 or 37 °C. While the cells grown in LB at 37°C displayed severe filamentation, as discussed in previous sections, the cells grown at 22°C displayed normal cell growth (Figure 5B bottom panels). We further verified the stable production of YpsA in slow growing cells by performing western blotting of cells harvested at mid-log phase and stationary phase (Figure S3D). Based on these results, it appears that YpsA is a growth rate-dependent cell division inhibitor.

A known factor that inhibits cell division depending on the presence of glucose is UgtP, which is a UDP-glucose diacylglycerol glucosyltransferase (Weart et al., 2007). Therefore, we tested if the YpsA-mediated filamentation is dependent on UgtP using an *ugtP* null strain. As shown in Figure S4, cells lacking *ugtP* also undergo filamentation upon increased production of YpsA suggesting that cell division inhibition by YpsA is independent of UgtP.

### Identification of Amino Acid Residues Important for YpsA Function

**Figure 6:**
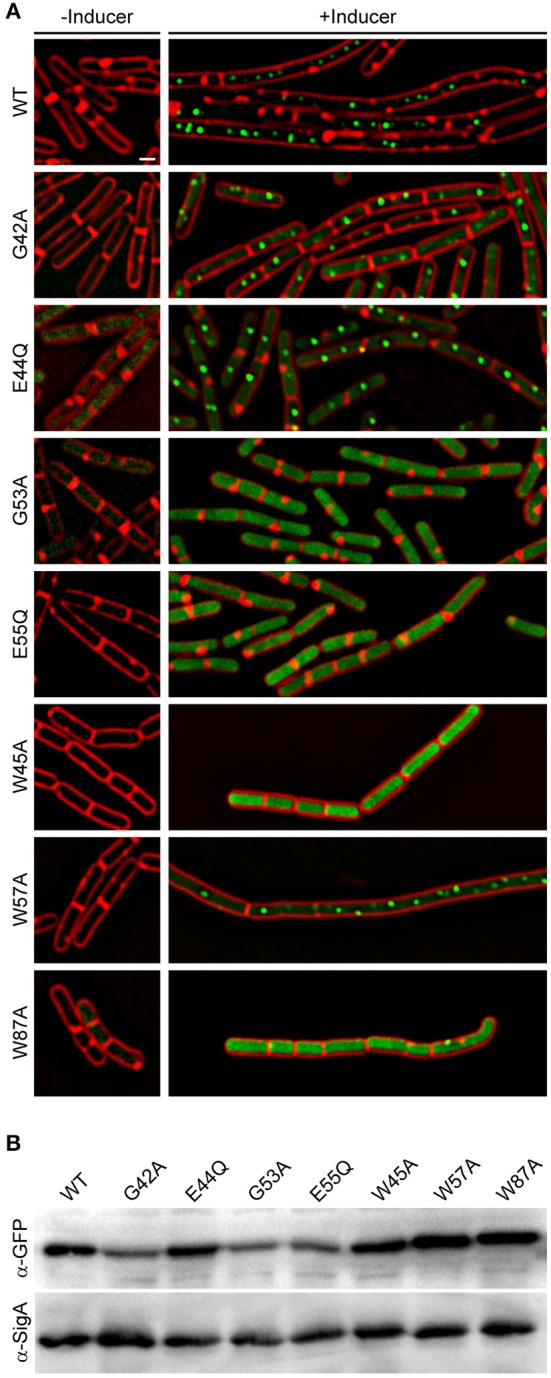
Site-directed mutagenesis reveals key residues in YpsA. **(A)** Cell morphologies of YpsA-GFP (WT; GG83) and GFP-fusions of G42A (RB119), E44Q (RB115), G53A (RB120), E55Q (RB116), W45A (RB35), W57A (RB26), and W87A (RB37) are shown. The cells were grown either in the absence (left panels) or presence (right panels) of inducer IPTG. Fluorescence of membrane stain FM4-64 (red) and GFP (green) are shown. Scale bar: 1 μm. **(B)** Production of GFP-tagged YpsA variants were detected by immunoblot of cell extracts of strains shown in **(A)** grown in the presence of inducer using anti-GFP and corresponding anti-SigA (loading control) antisera.

Aided by the crystal structure and computational analysis of the YpsA family of SLOG domains, we identified several conserved residues that are predicted to be important for maintaining the function of YpsA (Figure 1B; see arrows). We performed site-directed mutagenesis of two of the key residues that were speculated to be involved in substrate binding and generated GFP-tagged *ypsA* variants G53A and E55Q. We also generated other mutants to more generally explore YpsA function including G42A, E44Q, W45A, W57A, and W87A. We ensured that all mutants were stably produced through immunoblotting (Figure 6B). Fluorescence microscopy-based examination revealed that all YpsA variants except W57A were unable to trigger filamentation upon overexpression (Figure 6A), suggesting that YpsA function is compromised in all of these mutants. We also noticed that G53A, E55Q, W45A, and W87A mutants displayed impaired ability to form foci. This is consistent with the observation that the first two of these mutations disrupt the conserved, predicted nucleotide-binding site of the YpsA family (Burroughs et al., 2015), and the latter two likely disrupt a key strand and helix of the Rossmannoid fold.

### Overproduction of YpsA Inhibits Cell Division in *S. aureus*

**Figure 7:**
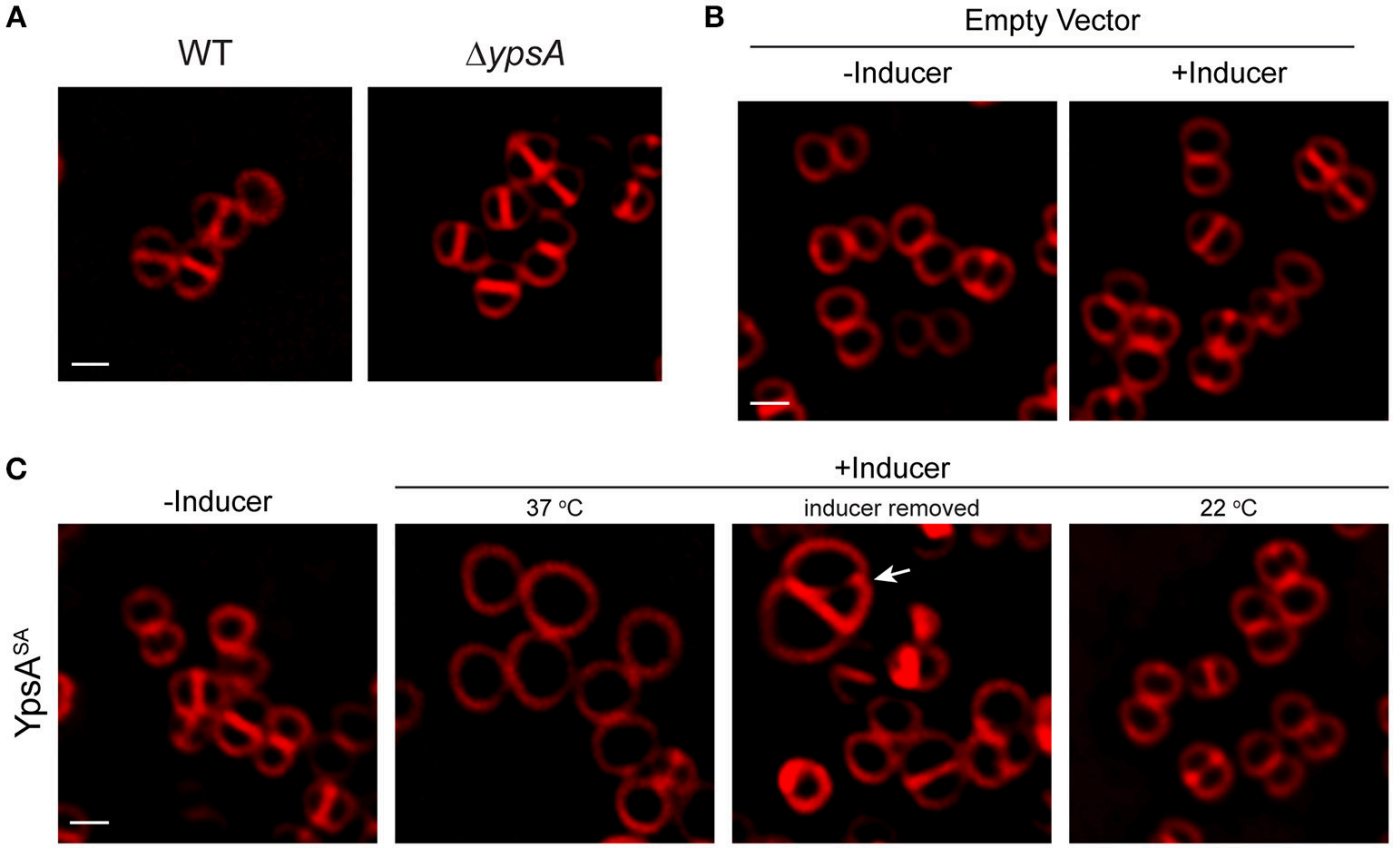
Production of YpsA^SA^ inhibits cell division in *S. aureus*. **(A)** Fluorescence micrographs of wild type (SH1000; left) or transposon-disrupted *ypsA* (RB162; right) strains. **(B)** Morphologies of SH1000 cells harboring empty vector (pEPSA5) in the absence of presence of inducer. **(C)** SH1000 cells harboring plasmid encoded xylose-inducible copy of *ypsA*^*SA*^ (pRB36) grown in the absence (left; 37°C) or presence of inducer grown at 37°C (second panel), or 3 h after removal of inducer (third panel), or when grown at 22°C (fourth panel) are shown. Arrow indicates a cell dividing unevenly. Cell membrane were visualized using FM4-64 dye (red). Scale bar: 1 μm.

To investigate if the role of YpsA is conserved in other Firmicutes, we chose to study the function of YpsA in *S. aureus*. Cells lacking intact *ypsA* in *S. aureus* (Fey et al., 2013), are viable and their cell morphology appear similar to WT control suggesting that, at least in the conditions tested here, *ypsA* is not an essential gene (Figure 7A). Next, we placed *S. aureus ypsA* (*ypsA*^*SA*^) under the control of a xylose-inducible promoter in a *S. aureus* plasmid vector. Cells capable of producing *ypsA*^*SA*^ or containing an empty vector control were then grown to mid-log phase (OD_600_ = 0.5) at 37°C at which point the inducer was added to produce YpsA^SA^ and culture samples for microscopy were harvested at the 1 h timepoint. As shown in Figure 7B, 1 h after the addition of inducer, the cell diameter of the WT control (0.88 ± 0.16 μm; *n* = 100; Figure 7A) and the vector control strain grown in the absence of inducer (0.88 ± 0.16 μm) and presence of inducer (0.89 ± 0.18 μm), resembled the inducible *ypsA*^*SA*^ strain grown in the absence of inducer (0.89 ± 0.17 μm; Figure 7C). Interestingly, cells overexpressing *ypsA*^*SA*^ were unable to undergo septation and displayed clear cell enlargement (1.72 ± 0.37 μm; *n* = 100; Figure 7C), a telltale sign of cell division inhibition in this organism. Therefore, the possible function of YpsA in inhibiting cell division is conserved in *S. aureus*, and perhaps in other Firmicutes which code for it despite the differences in their cell morphologies.

Next, we wondered whether the enlarged cells could revert back to normal growth upon removal of the inducer, similar to what was observed in *B. subtilis* in Figure 4. For this purpose, we centrifuged the cell culture and washed the inducer off the cell pellet with fresh medium three times and resuspended in fresh medium. Three hours after the removal of inducer, septation appears to have resumed, albeit with errors in septum positioning (see arrow in Figure 7C). The average diameter of the cells returned closer to WT-like (1.08 ± 0.35 μm; *n* = 100), indicating that enlarged cells overproducing YpsA could revert back to normal growth provided that the YpsA level is reduced. To test if cell division inhibition in *S. aureus* is also dependent on growth rate, we grew the inducible *ypsA*^*SA*^ strain in the presence of inducer at 22°C. Similar to what we observed in *B. subtilis* (Figure 5B), when *S. aureus* cells capable of overproducing YpsA^SA^ were grown in the presence of inducer at 22°C, we did not observe cell enlargement (0.92 ± 0.17 μm; *n* = 100; Figure 7C), suggesting that the phenomenon of growth rate-dependent cell division inhibition is conserved in *S. aureus* as well.

## Discussion

Bacterial cell division is a highly regulated process and many division factors have already been characterized especially in the model organisms *E. coli* and *B. subtilis*. Yet, cell division is only mildly affected even in the absence of a combination of known division regulators in these organisms (Monahan et al., 2014b), thus predicting the presence of other proteins that could affect the cell division process. Here, we discuss the role of YpsA, a protein of hitherto unknown function conserved in diverse Firmicutes. We show that YpsA offers protection against oxidative stress. However, the precise mechanism of how this is achieved remains to be elucidated. Next, we show that YpsA overproduction leads to impaired FtsZ ring assembly and ultimately cell division inhibition—although this could potentially be an indirect effect.

It has been reported that the *cotD-ypsA* transcriptional unit is repressed by the regulator essential for entry into sporulation, Spo0A (Fujita and Losick, 2003), which binds to a region upstream of *cotD* (Molle et al., 2003). It has been shown that *cotD* is also repressed by a late stage sporulation-specific transcriptional regulator, SpoIIID (Halberg and Kroos, 1994). Both *cotD* and *ypsA* transcripts are at similar levels in various growth conditions except in those that promote sporulation [Figure S1; (Nicolas et al., 2012; Zhu and Stülke, 2018)]. The function of CotD during normal growth, if any, needs to be evaluated. It has been reported that *cotD* level increases in a concentration-dependent manner in response to antibiotic treatment (Lin et al., 2005). In this report we show that cells lacking *ypsA* or overexpressing *ypsA* show no obvious sporulation defect and that YpsA-mediated cell division inhibition is dependent on the growth rate. Other reports exist that describe the connection between nutrient availability, growth rate, and cell division (Vadia and Levin, 2015; Westfall and Levin, 2017). At this time, we cannot rule out the involvement of regulatory mechanisms that affect cell division indirectly through ClpX-mediated (Camberg et al., 2009; Haeusser et al., 2009; Männik et al., 2018), or *E. coli* GidA-like (Lies et al., 2015), analogous systems.

YpsA mutants generated based on the crystal structure and sequence analysis revealed the importance of certain key residues for YpsA function (Figures 1B, 6). Interestingly, G53 and E55 of *B. subtilis* YpsA which form a conserved signature GxD/E motif, are predicted to be important for substrate-binding in the YpsA clade of proteins in the SLOG superfamily [Figure 1B; (Burroughs et al., 2015)]. Since foci-formation was disrupted in both G53A and E55Q mutants, it is plausible that substrate-binding allows for multimeric complex formation. It is noteworthy that mutants such as G42A and E44Q which are able to form foci, and therefore likely bind substrate, lack the ability to elicit filamentation. Similarly, YpsA-GFP overproducing cells grown in certain growth conditions were able to form foci but unable to induce filamentation (Figure 5). Assuming foci formation is indicative of substrate binding, these observations support a model in which substrate binding by YpsA is a prerequisite for cell division inhibition. However, substrate binding alone is not sufficient to induce filamentation.

The connection between NAD or its derivative ADP-ribose and the members of SLOG superfamily of proteins that belong to YpsA clade has been previously suggested (Burroughs et al., 2015). Given that ADP-ribosylation affects FtsZ polymerization (Ohashi et al., 1999; Ting et al., 2018), and YpsA is in close association with biological conflict systems and phosphoribosyl transferases (Figure 1B), it is possible that YpsA-mediated inhibition of cell division may involve ADP-ribosylation. Similarly, oxidative stress protection provided by YpsA might involve sensing or binding NAD or its derivatives as well. The link between metabolism of nicotinamide nucleotide, glucose availability, and oxidative stress has been reported previously (Imlay and Linn, 1988; Brumaghim et al., 2003).

Lastly, we show that overproduction of YpsA in another Firmicute, *S. aureus*, results in cell enlargement, indicative of cell division inhibition, specifically in a growth rate dependent fashion. This hints at a possible conserved role in cell division for YpsA in these Gram-positive organisms. In *B. subtilis*, a prophage associated protein of unknown function, YoqJ, also belongs to the YpsA family (Figure 1B). Given that there are clear examples of phage proteins affecting bacterial cell division (Zhou and Lutkenhaus, 2005; Ballesteros-Plaza et al., 2013; Kiro et al., 2013; Haeusser et al., 2014), it would be interesting to see if YoqJ also influences cell division. Although the GxD/E motif is conserved in YoqJ, several residues we identified to be essential for the cell division function of YpsA are not conserved in YoqJ (Figures 1B, 6). The Firmicutes-specific conserved gene coupling between *ypsA* and *gpsB* starkly contrasts the diversity of the gene neighborhoods found in other branches of the YpsA family phylogenetic tree. Superposition of conserved gene-neighborhoods onto the phylogenetic tree (Figure 1A) revealed a stark compartmentalization in conserved genome contexts. The *ypsA* and *gpsB* gene coupling is found only in one of the four major branches in the tree. Each of the other three branches displays distinct conserved neighborhood proclivities including: (1) a branch where YpsA couples strongly in a gene pair relationship with a phosphoribosyltransferase (PRTase) domain, (2) a branch where YpsA is found in scattered associations with various components of NAD processing and salvage pathways, and (3) a diverse collection of contexts across a broad class of bacterial lineages representative of the aforementioned nucleotide-centered biological conflict systems, where YpsA is likely to act in nucleotide signal-generation or nucleotide-sensing [Figure 1A; (Burroughs et al., 2015)]. These observations suggest that the *B. subtilis* YpsA may have acquired a more institutionalized role in cell division within the Firmicutes phylum. Nevertheless, understanding the precise biochemical mechanism by which *B. subtilis* YpsA executes its function would potentially shed light on the more general function of YpsA across a wide range of organisms and biological conflict systems.

## Materials and Methods

### Strain Construction and General Methods

All *B. subtilis* strains used in this study are isogenic derivatives of PY79 (Youngman et al., 1984). See Table S1A for strain information. Overproduction of YpsA was achieved by PCR amplifying *ypsA* using primer pairs oP106/oP108 (see Table S1B for oligonucleotide information) and ligating the fragment generated cut with SalI and NheI with IPTG-inducible *amyE* locus integration vector pDR111 (D. Rudner) also cut with SalI and NheI and the resulting plasmid was named pGG27. To construct a GFP fusion, *ypsA* fragment that was amplified with primer pairs oP106/oP107 and digested with SalI and NheI was ligated with *gfp* fragment generated with oP46/oP24 and cut with NheI/SphI and cloned into pDR111 digested with SalI/SphI, resulting in plasmid pGG28. The G42A, E44Q, W45A, G53A, E55Q, W57A, and W87A mutations were introduced using the QuikChange site-directed mutagenesis kit (Agilent) using pGG28 as template. The strain expressing *ypsA-gfp* under the control of its native promoter from an ectopic locus was constructed by PCR amplifying *ypsA* and its promoter region using the primer pairs oP301/oP107 and by digesting the resulting PCR product with HindIII and NheI. The *gfp* fragment was PCR amplified using the primer pairs oP46/oP47 and digested with NheI and BamHI. The digested products were then ligated into the *amyE* integration plasmid pDG1662 cut with HindIII and BamHI, and the resulting plasmid was named pRB43. *ypsA-3xflag* was constructed via two step PCR using pGG27 as a template. Round one PCR was completed using primers oP106 and oP291. The PCR product from round one was then used as a template for round two PCR, which was completed using primer pairs oP106 and oP292. The final PCR product was then cloned into pDR111 using SalI and NheI restriction sites, making plasmid pRB33. Similarly, *ypsA-gfp-3xflag* was constructed via two step PCR using pGG28 as a template. Round one PCR was completed using primers oP106 and oP349. The PCR product from round one was then used as a template for round two PCR, which was completed using primers oP106 and oP350. The final PCR product was then cloned into pDR111 using SalI and SphI restriction sites, making plasmid pRB34. The engineered plasmids were then used to introduce genes of interest via double crossover homologous recombination into the *amyE* locus of the *B. subtilis* chromosome. To produce *S. aureus* YpsA in *S. aureus* strain SH1000, *ypsA*^*SA*^ fragment (PCR amplified with oRB27/oP314 primer pairs) was cloned into xylose-inducible pEPSA5 plasmid using EcoRI and BamHI restriction sites (Forsyth et al., 2002), generating plasmid pRB36. Plasmids were first introduced into *S. aureus* RN4220 via electroporation, and then transduced into SH1000 (Eswara et al., 2018).

### Media and Culture Conditions

Overnight *B. subtilis* cultures grown at 22°C in Luria-Bertani (LB) growth medium were diluted 1:10 into fresh LB medium and grown in 37°C to mid-logarithmic growth phase (OD_600_ = 0.5), unless otherwise stated. Expression of genes under IPTG-controlled promoter was induced by addition of 250 μM IPTG (final concentration) to the culture medium at mid-log phase unless noted otherwise. Overnight *S. aureus* cultures were grown at 22°C in tryptic soy broth (TSB) supplemented with 15 μg/ml chloramphenicol and/or 5 μg/ml erythromycin where required for plasmid maintenance. Cultures were then diluted 1:10 into fresh medium containing appropriate antibiotics and grown to mid-logarithmic growth phase (OD_600_ = 0.5), unless otherwise stated. Expression of genes under xylose-controlled promoter was induced by the addition of 1% xylose when required.

### Sporulation Assay

Sporulation assay was conducted using resuspension protocol as described previously (Eswaramoorthy et al., 2009). Briefly, overnight cultures of *B. subtilis* cells were grown in LB medium at 22°C, were diluted 1:10 in fresh casein hydrolysate medium (CH, KD Medical) and grown in 37°C to mid-log phase twice before culture was resuspended in Sterlini-Mandelstam sporulation medium (SM, KD Medical) to induce sporulation (Sterlini and Mandelstam, 1969). Growth in CH medium and entry into sporulation in SM medium were monitored via fluorescence microscopy. Total viable cell counts (CFU/ml prior to heat treatment) and spore counts (CFU/ml after incubation at 80°C for 10 min) were obtained for calculating sporulation frequency (spore count/viable count).

### Disc Diffusion Assay

All disc diffusion assays were completed on LB agar plates. Strains PY79 and RB42 were grown until OD_600_ = 0.5, and 100 μl of each culture was spread on the surface of LB plates using sterile glass beads. After the plates were dry, 15 μl of 0.1 mM mitomycin C (Alfa Aesar), 1% SDS (Fisher BioReagents), 5 mg/ml daptomycin (Biotang), different concentrations of ethanol (Fisher BioReagents), and a range of concentrations of hydrogen peroxide (Fisher Chemical) was added to 7 mm Whatman filter paper discs, which were then placed equidistant from each other on top of the inoculated plate. Additionally, commercially available discs (Becton-Dickinson) containing 10 units of Penicillin or 30 μg of vancomycin was also used in this assay. Discs containing 15 μl of sterile water were used as our mock control. Plates were then incubated overnight at 37°C. The diameter of the discs (7 mm) was subtracted from the zone of inhibition measurements.

### Immunoprecipitation Assay

The YpsA-FLAG immunoprecipitation was performed using FLAGIPT1 immunoprecipitation kit (Sigma-Aldrich) as described previously (Eswaramoorthy et al., 2014). Briefly, 1 ml cell lysates of cells harvested from 20 ml LB culture induced with 250 μM IPTG (final concentration) grown for 2 h post-induction to produce FLAG-tagged proteins or untagged negative control were generated by sonication. Cell extracts were then incubated overnight with 50 μl anti-FLAG M2 affinity beads supplied by the manufacturer. The beads were then washed 3 times with 1x wash buffer and the supernatant was removed by pipetting. Proteins bound to the beads were stripped by adding 80 μl of 2x sample buffer supplied by the manufacturer and heating at 100°C for 5 min. Western blot using anti-Flag antibody (Invitrogen) was used to detect Flag-tagged proteins in all fractions collected.

### Microscopy

Aliqouts containing 1 ml of culture (*B. subtilis* and *S. aureus*) were washed in phosphate buffered saline (PBS) and then resuspended in 100 μl of PBS containing 1 μg/ml FM4-64 (membrane stain). For imaging, 5 μl of sample was then spotted onto a glass bottom dish (MatTek) and it was covered with an 1% agarose pad made with sterile water. Still imaging was completed at room temperature. For time-lapse microscopy, 5 μl aliquots of culture were spotted onto a glass bottom dish, and the sample was covered with 1% agarose pad made with LB culture medium. Agarose pads were supplemented with 2 μl of FM4-64 (1 μg/ml) and/or inducer were required to induce the expression of desired genes prior to data collection. Microscopy was performed using GE Applied Precision DeltaVision Elite deconvolution fluorescence microscope equipped with a Photometrics CoolSnap HQ2 camera and environmental chamber. Typically, 17 planes (Z-stacks) every 200 nm was acquired of all static image data sets and 5 planes every 200 nm was acquired for time-lapse microscopy to minimize phototoxicity. The images were then deconvolved using SoftWorx software provided by the manufacturer. Fluorescence intensity of GFP signal was quantified using the data inspector tool on the SoftWorx software. Briefly, fluorescence signal was measured in arbitrary units in cells producing GFP and background noise of equivalent area from a neighboring region devoid of cells or cell debris in the field of view was subtracted to acquire the final value.

### Sequence Analysis

YpsA sequence similarity searches were performed using the PSI-BLAST program (Altschul et al., 1997) against the non-redundant (NR) database of the National Center for Biotechnology Information (NCBI). Multiple sequence alignments were built by the MUSCLE and KALIGN programs (Edgar, 2004; Lassmann et al., 2009), followed by manual adjustments on the basis of profile–profile and structural alignments. Genes residing in conserved neighborhoods were identified through clustering carried out with the BLASTCLUST program (ftp://ftp.ncbi.nih.gov/blast/documents/blastclust.html). Phylogenetic analysis was conducted using an approximately-maximum-likelihood method implemented in the FastTree 2.1 program under default parameters (Price et al., 2010), and resulting trees were visualized initially in the FigTree program [http://tree.bio.ed.ac.uk/software/figtree/].

## Author Contributions

RB and PE designed the study. RB, MH, GG, SA, MW, and PE constructed strains and performed experiments. AB and LA conducted bioinformatics analysis. RB, MW, AB, LA, and PE analyzed data. RB and PE wrote the manuscript. All authors read and commented on the final manuscript.

### Conflict of Interest Statement

The authors declare that the research was conducted in the absence of any commercial or financial relationships that could be construed as a potential conflict of interest.
