# Supplementary material for: Deciphering the Role of a SLOG Superfamily Protein YpsA in Gram-Positive Bacteria
Source: Front Microbiol. 2019 Apr 5;10:623. doi: 10.3389/fmicb.2019.00623 (PMC6459960; doi:10.3389/fmicb.2019.00623)
Supplement: Supplementary file 2 [file Data_Sheet_1.pdf]

Supplemental Data

Deciphering the role of a SLOG superfamily protein YpsA in Gram-positive bacteria

Robert S. Brzozowski[1], Mirella Huber[1], A. Maxwell Burroughs[2], Gianni Graham[1], Merryck Walker[1], Sameeksha S. Alva[1], L. Aravind[2], and Prahathees J. Eswara[1,*]

[1]Department of Cell Biology, Microbiology and Molecular Biology, University of South Florida, Tampa, FL 33620, USA

[2]National Center for Biotechnology Information, National Library of Medicine, National Institutes of Health, Bethesda, MD 20894, USA

Figure S1. Transcript levels of *cotD*, *ypsA*, and *gpsB* in *B. subtilis* at various growth conditions (28, 29).

Figure S2. Immunoprecipitation assay. A strain co-producing YpsA-FLAG and YpsA-GFP under the control of IPTG (RB222) was induced at mid-log (OD_{600} =0.5). Cells were harvested 2 h post-induction and processed for anti-Flag immunoprecipitation as described in materials and methods section. Cell lysate (Load) and eluate fractions were subjected to immunoblotting with antisera specific to FLAG, GFP, or SigA.

Figure S3. (A) Growth curves (OD_{600}) of strains WT (PY79) and Δ*ypsA* (RB42) grown in LB medium at 37 °C and 22 °C, and in CH medium, followed for 5 h. (B) Growth curves of cells overproducing *ypsA* (GG82) or *ypsA-gfp* (GG83) grown in CH medium, with or without the supplementation of 1% glucose or 1% sucrose, monitored for 5 h. (C) Growth curves of strains *ypsA* (GG82) and *ypsA-gfp* (GG83) grown in LB medium at 22 °C or 37 °C, tracked for 5 h. All experiments were performed in triplicates and representative graphs are shown for (A), (B), and

(C). (D) Cells (1 ml culture; GG83) harvested at mid-log phase (OD_{600} =0.5) and 3 h post mid-log (stationary) were subjected to immunoblotting against antisera specific to GFP or SigA. Ratio of GFP/SigA (arbitrary units) are shown at the bottom.

Figure S4. Cell morphologies of inducible *ypsA* cells (GG82) grown in the absence (A) or presence (B) of inducer. Also shown are the cell morphologies of inducible *ypsA* in a strain lacking *ugtP* (RB212) grown in the absence (C) or presence (D) of inducer. Scale bar: 1 μm.

Table S1. Strains and oligonucleotides used in this study

Video S1. Timelapse of YpsA-GFP foci movement. DIC and GFP fluorescence information of strain GG83 producing YpsA-GFP imaged in the presence of inducer at 1-min interval for 10 min. Arrow indicates foci that move significantly.

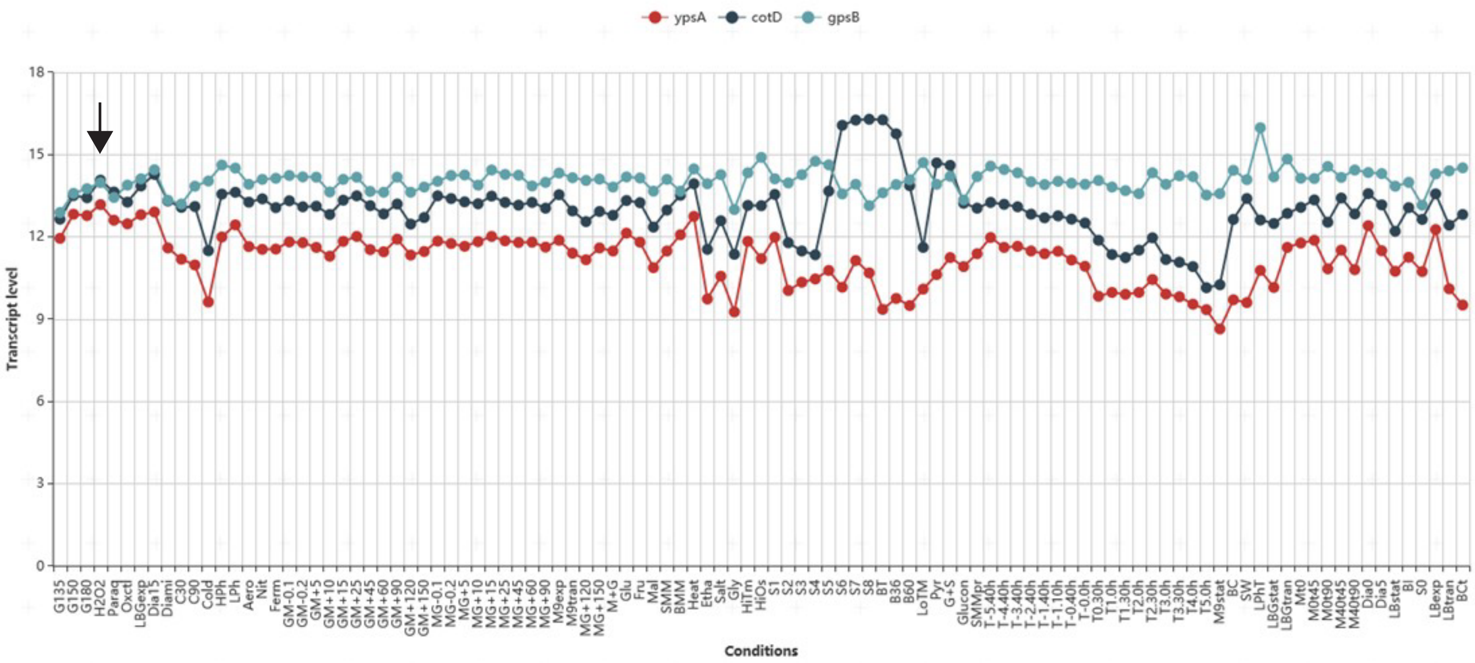
ypsA
cotD
gpsB
18
15
12
9
6
3
0
Transcript level
G135 G150 G180 H2O2 Paraq Oxctl LBGexp Dia15 Diami C30 C90 Cold HPh LPh Aero Nit Ferm GM-0.1 GM-0.2 GM+5 GM+10 GM+15 GM+25 GM+45 GM+60 GM+90 GM+120 GM+150 MG-0.1 MG-0.2 MG+5 MG+10 MG+15 MG+25 MG+45 MG+60 MG+90 M9exp M9tran MG+120 MG+150 M+G Glu Fru Mal SMM BMM Heat Etha Salt Gly HiTm HiOs S1 S2 S3 S4 S5 S6 S7 S8 BT B36 B60 LoTM Pyr G+S Glucon SMMpr T-5.40h T-4.40h T-3.40h T-2.40h T-1.40h T-1.10h T-0.40h T-0.0h T0.30h T1.0h T1.30h T2.0h T2.30h T3.0h T3.30h T4.0h T5.0h M9stat BC SW LPhT LBGstat LBGtran Mt0 M0t45 M0t90 M40t45 M40t90 Dia0 Dia5 LBstat BI S0 LBexp LBtran BCt
Conditions

Figure S1

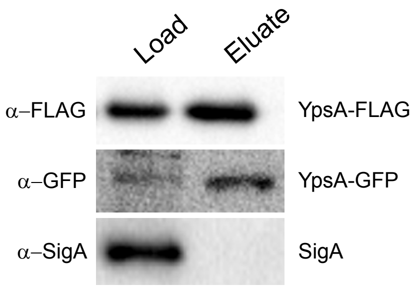

Figure S2

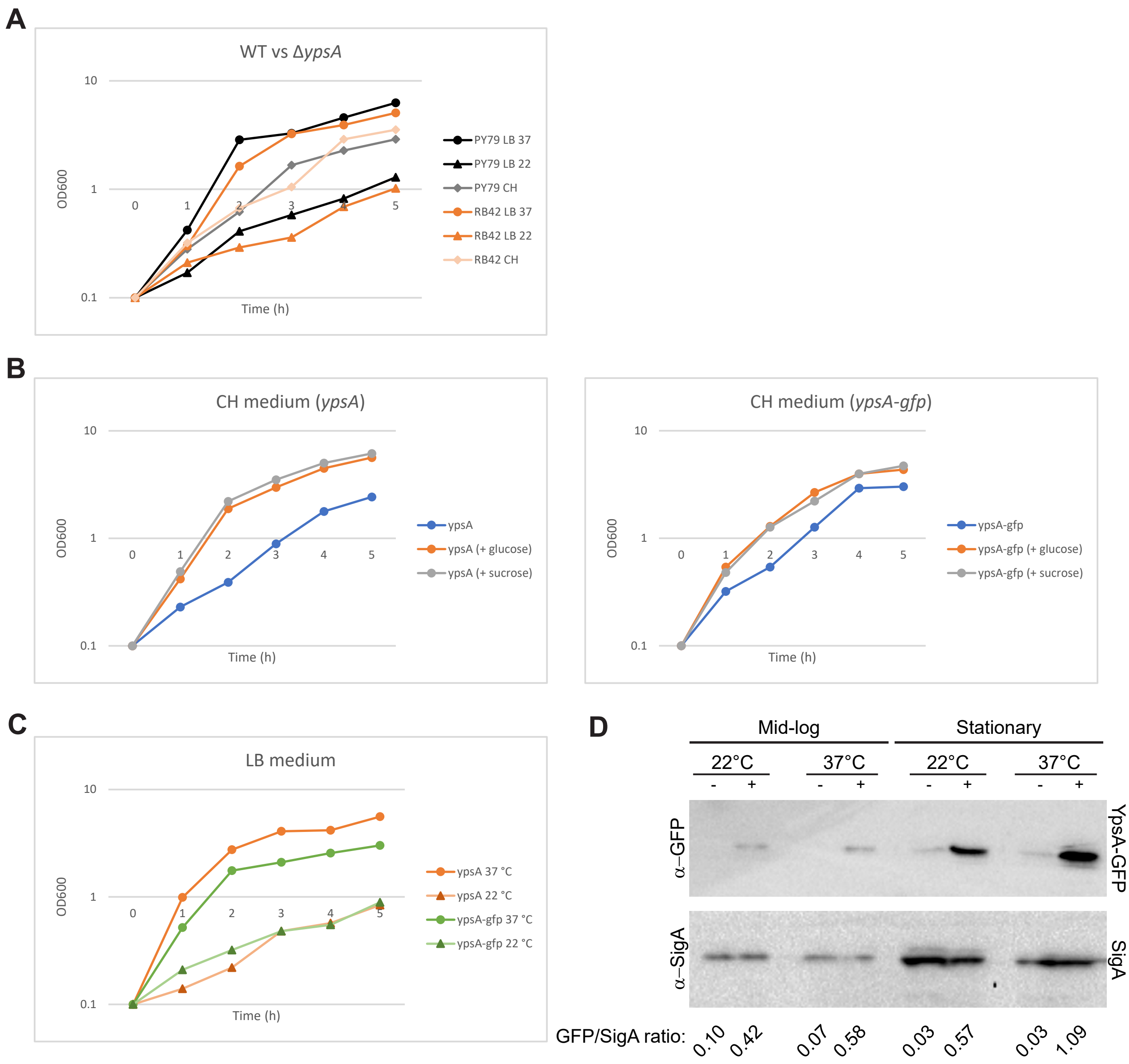
A
WT vs ΔypsA
10
1
0.1
OD600
0
1
2
3
4
5
Time (h)
PY79 LB 37
PY79 LB 22
PY79 CH
RB42 LB 37
RB42 LB 22
RB42 CH
B
CH medium (ypsA)
ypsA
ypsA (+ glucose)
ypsA (+ sucrose)
CH medium (ypsA-gfp)
ypsA-gfp
ypsA-gfp (+ glucose)
ypsA-gfp (+ sucrose)
C
LB medium
ypsA 37 °C
ypsA 22 °C
ypsA-gfp 37 °C
ypsA-gfp 22 °C
D
Mid-log
Stationary
22°C
37°C
22°C
37°C
-
+
-
+
-
+
-
+
α–GFP
YpsA-GFP
α–SigA
SigA
GFP/SigA ratio:
0.10
0.42
0.07
0.58
0.03
0.57
0.03
1.09

Figure S3

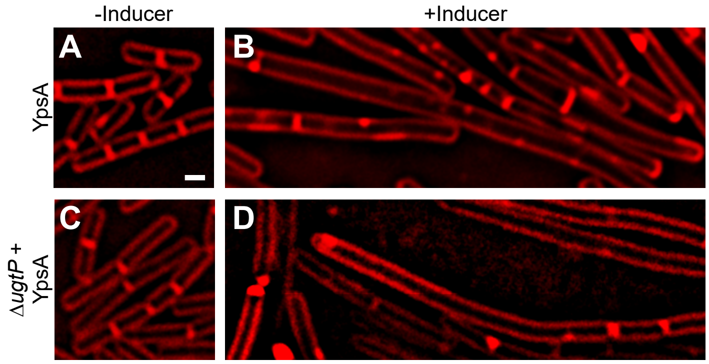

Figure S4

Table S1 Strains and oligonucleotides used in this study

Strains used in this study

Species	Strain	Genotype	Reference
B. subtilis	PY79	Wild type	Youngman *et al.* (1984)
B. subtilis	RB42	Δ*ypsA* ::erm	Derived from BKE22190 (BGSC*)
B. subtilis	GG82	*amyE* ::*P* $_{hyperspank}$ -*ypsA* ^{Bs} *spec*	This study
B. subtilis	GG83	*amyE*::*P* $_{hyperspank}$ -*ypsA* ^{Bs} -*gfp spec*	This study
B. subtilis	RB95	*amyE*::*P* $_{hypersank}$ -*ypsA* ^{Bs} -*mCherry spec*	This study
B. subtilis	RB43	Δ*gpsB* ::*tet amyE*::*P* $_{hyperspank}$ -*ypsA* ^{Bs} *spec*	This study
B. subtilis	RB44	Δ*gpsB* ::*tet amyE*::*P* $_{hyperspank}$ -*ypsA* ^{Bs} -*gfp spec*	This study
B. subtilis	PE92	*ftsAZ* ::*ftsAZ-gfp* Ωerm	Eswaramoorthy *et al.* (2011)
B. subtilis	RB15	*ftsAZ*::*ftsAZ-gfp*Ω*erm amyE*::*P* $_{hyperspank}$ -*ypsA* ^{Bs} *spec*	This study
B. subtilis	RB97	*ftsAZ*::*ftsAZ-gfp*Ω*erm amyE*::*P* $_{hyperspank}$ -*ypsA* ^{Bs} -*mCherry spec*	This study
B. subtilis	RB119	*amyE* ::*P* $_{hyperspank}$ -*ypsA* $^{Bs\text{-}\ G42A}$-gfp *spec*	This study
B. subtilis	RB115	*amyE* ::*P* $_{hyperspank}$ -*ypsA* $^{Bs\text{-}\ E44Q}$-gfp *spec*	This study
B. subtilis	RB35	*amyE* ::*P* $_{hyperspank}$ -*ypsA* $^{Bs\text{-}\ W45A}$-gfp *spec*	This study
B. subtilis	RB120	*amyE* ::*P* $_{hyperspank}$ -*ypsA* $^{Bs\text{-}\ G53A}$-gfp *spec*	This study
B. subtilis	RB116	*amyE* ::*P* $_{hyperspank}$ -*ypsA* $^{Bs\text{-}\ E55Q}$-gfp *spec*	This study
B. subtilis	RB26	*amyE* ::*P* $_{hyperspank}$ -*ypsA* $^{Bs\text{-}\ W57A}$-gfp *spec*	This study
B. subtilis	RB37	*amyE*::*P* $_{hyperspank}$ -*ypsA* $^{Bs\text{-}W87A}$ -*gfp spec*	This study
B. subtilis	RB121	*amyE*::*P* $_{hyperspank}$ -*ypsA* ^{Bs} -*flag spec*	This study
B. subtilis	RB125	*amyE*::*P* $_{hyperspank}$ -*ypsA* ^{Bs} -*gfp-flag spec*	This study
B. subtilis	RB160	Δ*ypsA*::*erm*; *amyE*::*P* $_{hyperspank}$ -*ypsA* ^{Bs} *spec*	This study; derived from BKE22190 (BGSC*)
B. subtilis	RB161	Δ*ypsA*::*erm*; *amyE*::*P* $_{hyperspank}$ -*ypsA* ^{Bs} -*gfp spec*	This study; derived from BKE22190 (BGSC*)
B. subtilis	RB212	Δ*ugtP*::*erm amyE*::*P* $_{hyperspank}$ -*ypsA* ^{Bs} *spec*	Derived from BKE21920 (BGSC*)
B. subtilis	RB221	*amyE*::*P* $_{ypsA}$ -*ypsA* ^{Bs} -*gfp cat*	This study
B. subtilis	RB222	*bkdB*::*Tn917*:: *amyE*::*P* $_{hyperspank}$ -*ypsA-3xflag spc*; *amyE*::*P* $_{hyperspank}$ -*ypsA-gfp spc*::*erm*	This study; bkdB::Tn917::amyE::cat (Amy Camp)
S. aureus	PL3055	Wild type SH1000	Eswara *et al.* (2018)
S. aureus	RB162	*ypsA* ::tn *erm* (SH1000 background)	This study; derivative of NE1697 - Fey *et al.* (2013)
S. aureus	RB143	SH1000 pEPSA5 cat	This study
S. aureus	RB128	SH1000 pRB36 (pEPSA5 backbone, *P* $_{xyl}$ -*ypsA* ^{Sa} *cat*)	This study

*BGSC - Bacillus Genetic Stock Center

Oligonucleotides used in this study

Primer	Sequence (5' to 3')
oP24	GCCGCATGC*TTA* TTTGTATAGTTCATCCATGCC
oP46	AAAGCTAGCATGAGTAAAGGAGAAGAACTTTTC
oP47	AAAGGATCC*TTA* TTTGTATAGTTCATCCATGCC
oP106	AAAGTCGAC*ACATAAGGAGGAACTACT* ATGAAAGTATTGGCAATAACGGGCTATAAACCG
oP107	AAAGCTAGCGTAGCTGTCTTCCTCCACTGTCACTCTCAAGTCATC
oP108	AAAGCTAGC*TTA* GTAGCTGTCTTCCTCCACTGTCACTCTCAAGTCATC
oP168	AAAGCTAGCATGGTTTCCAAGGGCGA
oP169	AAAGCATGC*TTA* TTTGTACAGCTCATC
oP291	ATAATCACCATCATGATCCTTATAATCGTAGCTGTCTTCCTCCACTGTCACTCTCAAGTC
oP292	AATAAGCTAGC*TTA* CTTGTCGTCATCGTCTTTGTAGTCGATATCATGATCCTTATAATCACCATCATGATCCTTATAATC
oP301	AATAAAAGCTTGAGGAAATATTCCTCTTAAATATTCCCCGGGAAAGCGC
oP314	AATAAGGATCCTTAGAAACTTTGATCTTCAGACCACTGTAAGTC
oP349	ATCATGATCCTTATAATCACCATCATGATCCTTATAATCTTTGTATAGTTCATCCATGCC
oP350	AATAAGCATGC*TTA* CTTGTCGTCATCGTCTTTGTAGTCGATATCATGATCCTTATAATC
oRB9	GCCTCTAGA*AATAATTTTGTTTAACTTTAAGAAGGAGATATA* ATGAAGTATTGGCAATA
oRB11	ATTGCTTTTTTGGATGAAGGATTAGAAGCGATTTTAATTTCGGGCCAGCTTGGAGTTGAG
oRB12	CTCAACTCCAAGCTGGCCCGAAATTAAAATCGCTTCTAATCCTTCATCCAAAAAAGCAAT
oRB13	ATTTCGGGCCAGCTTGGAGTTGAGCTGGCGGCGGCAGAAGCTGCATATGATTTGCAG
oRB14	CTGCAAATCATATGCAGCTTCTGCCGCCGCCAGCTCAACTCCAAGCTGGCCCGAAAT
oRB15	CCATTTTACGAGCAGGAAAAGAACGCGAAAGAACCGAATAAAGAACAGTATGAAGCAGTTCTG
oRB16	CAGAACTGCTTCATACTGTTCTTTATTCGGTTCTTTCGCGTTCTTTTCCTGCTCGTAAAA
oRB27	AAAGAATTC*TAATGAGGTGGAAAAA* ATGGTTAAAACAGTTTATGTAACAGGTTACAAATCA
oRB33	GCCGGATCC*TTA* ATGGTGATGGTGATGGTGGTAGCTGTCTTCCTCCACTGT
oRB34	CTGATTGCTTTTTTGGATGAAGCATTAGAATGGATTTTAATTTCG
oRB35	CGAAATTAAAATCCATTCTAATGCTTCATCCAAAAAAGCAATCAG
oRB36	ATTTTAATTTCGGGCCAGCTTGCAGTTGAGCTGTGGGCGGCAGAAGCTGCA
oRB37	TGCAGCTTCTGCCGCCCACAGCTCAACTGCAAGCTGGCCCGAAATTAAAAT
oRB38	CTGATTGCTTTTTTGGATGAAGGATTACAATGGATTTTAATTTCG
oRB39	CGAAATTAAAATCCATTGTAATCCTTCATCCAAAAAAGCAATCAG
oRB40	ATTTTAATTTCGGGCCAGCTTGGAGTTCAGCTGTGGGCGGCAGAAGCTGCA
oRB41	TGCAGCTTCTGCCGCCCACAGCTGAACTCCAAGCTGGCCCGAAATTAAAAT
